# The Canine Sacroiliac Joint: 2. Common Surface Variants

**DOI:** 10.1111/ahe.70159

**Published:** 2026-07-27

**Authors:** Janek Gensicke, Mirjam Kalusa, Thomas Grochow, Rebecca L. Tartsch, Alexandra S. Klick, Christoph K. W. Mülling, Simone A. Fietz, Nicole Röhrmann

**Affiliations:** ^1^ Institute of Veterinary Anatomy, Histology and Embryology Leipzig University Leipzig Germany

**Keywords:** anatomy, animals, arthrology, dogs, hyaline cartilage, sacroiliac joint

## Abstract

The canine sacroiliac joint (CSIJ) is crucial for transferring load between the axial skeleton and the pelvic limbs. However, the morphology of this joint beyond the primary articulation is not well characterized. This second study on the CSIJ aimed to systematically describe common morphological variants, including synovial surface area (SSA) shape types, non‐fused ossification centers (NFOC), and accessory sacroiliac joints (ASIJ), and to assess their morphometric characteristics and potential biomechanical relevance. A total of 29 canine pelves (*n* = 58 CSIJ) were examined. SSA morphology was categorized, NFOC were identified based on characteristic surface interruptions of the articular surface, and ASIJ were documented in terms of frequency, localization, and size. Seven distinct SSA shape types were identified, with auricular, bifoliate, and phrygian cap–like configurations being the most prevalent. Only one‐third of the examined joints exhibited the commonly described auricular or crescent shape. NFOC were present in 27.60% of CSIJ and occurred predominantly in dogs with a crown–rump length (CRL) exceeding 740 mm. Accessory articulations were detected in 74.10% of CSIJ. These were frequently bilateral and, in some cases, presented as multiple structures within a single joint. The ASIJ represented independent diarthrodial joints lined with hyaline cartilage. Their additional articular surface areas (AASA) correlated with body size and overall CSIJ dimensions. In conclusion, the examined CSIJ specimens demonstrated considerable anatomical variability extending beyond the primary articular surfaces. Variations in SSA morphology, the presence of NFOC, and the occurrence of ASIJ appear to represent frequent physiological variants rather than rare anomalies. Recognition of these features is essential for accurate anatomical characterization and imaging interpretation and provides a structural basis for future biomechanical and clinical investigations of the CSIJ.

## 1 Introduction

The canine sacroiliac joint (CSIJ) constitutes a central component of the lumbopelvic region, mediating mechanical interactions between the axial skeleton and the pelvic limbs (Breit and Künzel 2001b; Dyce et al. 2010; Frewein et al. 2004; Salomon 2020). Its biomechanical function is intrinsically linked to joint morphology and surface configuration. Consequently, distinguishing physiological variants from pathological alterations is of fundamental importance (Standring et al. 2021).

Breed‐specific differences in pelvic and lumbosacral joint morphology have been described. More sagittal oriented sacroiliac joints, as observed in German Shepherd Dogs, are associated with increased craniocaudal translation compared with the more oblique joint orientation described in breeds such as Rottweilers (Breit and Künzel 2001a). These morphological characteristics may contribute to the development of early‐onset CSIJ alterations (Gembardt 1974; Gregory et al. 1986).

Developmental aspects further contribute to the morphological variability of the CSIJ. In large‐breed dogs, incomplete fusion of ossification centers (OC) within the sacral wing has been documented, persisting into adulthood as discrete defects or concavities of the sacral synovial surface area (SSA). Interestingly, such concavities may enhance local joint interlocking and reduce craniocaudal shear forces, thereby potentially exerting a stabilizing effect in otherwise mechanically disadvantaged joints (Breit and Künzel 2001b). However, these features can sometimes be misinterpreted as pathological lesions in diagnostic imaging (Götz et al. 1993; Prassopoulos et al. 1999).

Beyond intraspecific variation, comparative anatomical studies across species provide additional evidence for distinct morphological variants of the sacroiliac joint (SIJ). In this context, SSA corresponds to the auricular surface, reflecting its auricular‐ or crescent‐shaped morphology (Evans and Lahunta 2013). Although this terminology is standardized, the detailed morphology of SSA exhibits pronounced inter‐ and intra‐species variability, with distinct shape types in cats, horses, and humans (Dalin and Jeffcott 1986; Jesse et al. 2017; Pallandre et al. 2020). These variations have been associated with differences in stress distribution and pain prevalence (Jesse et al. 2017).

In humans, several SIJ variants have been identified based on computed tomography (CT) imaging, including non‐fused ossification centers (NFOC) and accessory sacroiliac joints (ASIJ). The latter represent additional articulations located dorsal to the main joint and have been strongly associated with degenerative changes (Demir et al. 2007; El Rafei et al. 2018), as well as with low back pain (Hadley 1950; Kang et al. 2017; Prassopoulos et al. 1999; Rosa Neto et al. 2009).

In summary, SIJ morphology is highly variable and closely linked to joint mechanics, stability, and the development of clinical disease across species. Therefore, a comprehensive understanding of the morphological spectrum of the CSIJ is essential for the accurate interpretation of imaging findings and for elucidating potential predispositions to joint pathology.

Accordingly, this study aims to:

1. characterize SSA shape variations, and
2. identify and quantify developmental ossification anomalies (including NFOC and ASIJ)

## 2 Materials and Methods

This study was conducted using the same cohort of 29 canine specimens that were previously subjected to histological and morphometric analyses of the CSIJ (Table S1). The same dissection protocol was applied. It was evaluated and approved by the Ethical Committee of the Faculty of Veterinary Medicine, Leipzig University (EK 7/2021, 3 May 2021; Gensicke et al. 2026).

From each dog, paired CSIJ were obtained and subdivided into four joint portions: left iliac (LI), left sacral (LS), right iliac (RI), and right sacral (RS).

All CSIJ underwent macroscopic morphological assessment and were documented photographically using an iPhone 14 Pro (Apple Inc., Cupertino, CA, USA; 48‐megapixel sensor; maximum image resolution: 8064 × 6048 pixels). Each joint portion (*n* = 116) was evaluated for SSA shape types, the presence of NFOC of the sacral SSA, and ASIJ.

### 2.1 Morphological Analysis

The frequency of each morphological feature was recorded for each joint as well as for each individual. Side‐related distribution patterns were assessed by comparing left and right CSIJ, and the symmetry of NFOC and ASIJ was evaluated in specimens presenting bilateral findings.

Articulation characteristics—including surface texture, shape, curvature, and iliac–sacral congruency—were assessed. The classification of shape types was primarily guided by the classification proposed by Pallandre et al. (2020). In cases of ASIJ, anatomical localization was documented relative to the respective iliac and sacral joint portions.

### 2.2 Morphometric Features of Common Surface Variants

Morphometric analysis of SSA, NFOC, and ASIJ was performed using the same two‐dimensional method described previously in Gensicke et al. (2026).

### 2.3 Histology of ASIJ Cartilage

A histological evaluation of the caudodorsal ASIJ cartilage was performed on all four joint portions (LI, LS, RI, RS) of dog no. 25 (Table S1), as this was the only fresh specimen available. Initially, the entire articular surface was fixed in 4% buffered formalin (Carl Roth GmbH & Co. KG, Karlsruhe, Germany). The surrounding osseous tissue was subsequently decalcified and progressively softened using an ultrasonic bath under the influence of Chelaplex (ethylenediaminetetraacetic acid (EDTA) and tris(hydroxymethyl)aminomethane) (Dr. K. Hollborn & Söhne GmbH & Co. KG, Leipzig, Germany) until the superficial cartilage layer could be readily separated. The samples were embedded in paraffin blocks, and a single sagittal section approximately 1 μm thick was obtained from each sample. The four sections were stained with haematoxylin and eosin (H&E) (Carl Roth GmbH & Co. KG, Karlsruhe, Germany). Haematoxylin stains basophilic structures, particularly nucleic acids within the cell nuclei and the nucleolus (DNA and RNA), in shades of blue to purple, whereas eosin stains eosinophilic structures such as cytoplasmic proteins and extracellular matrix components in various shades of pink (Mescher 2018). Microscopic examination was performed using a light microscope (Axioscope 5, Carl Zeiss Microscopy GmbH, Oberkochen, Germany) equipped with an Axiocam 208 colour camera (Carl Zeiss Microscopy GmbH, Oberkochen, Germany) and Zen lite software (Version 3.13; Carl Zeiss Microscopy GmbH, Oberkochen, Germany).

Tissue types were identified and differentiated based on established histomorphological criteria, including cellular composition and extracellular matrix characteristics. Cartilaginous tissues were identified by the presence of chondrocytes within lacunae and the characteristic organization of the cartilage matrix. Fibrocartilage was characterized by a collagen‐rich matrix with chondrocytes frequently arranged in rows. Hyaline cartilage was characterized by a homogeneous matrix containing rounded chondrocytes, occasionally forming isogenous groups. In contrast, elastic cartilage contained a dense network of elastic fibres within the matrix, with chondrocytes likewise in lacunae and displayed a more flexible, resilient structure compared to hyaline cartilage (Liebich 2012).

All images were digitized according to the protocol described in our previous study (Gensicke et al. 2026).

### 2.4 Statistical Analysis of ASIJ

Measurements of additional articular surface areas (AASA) were compiled in Microsoft Excel 2016 (Microsoft Corporation, Redmond, WA, USA) and analysed using jamovi (version 2.6.44.0, The jamovi project, Sydney, Australia). Normality was assessed using the Shapiro–Wilk test (*p* < 0.05). As AASA data were not normally distributed, nonparametric methods were applied throughout, including the Wilcoxon signed‐rank test for paired comparisons and Spearman's rank correlation coefficient for correlation analyses (*p* < 0.05).

AASA sizes were summarized descriptively per dog, per joint portion, and per anatomical location. For inferential analyses, AASA values were summed per joint portion to account for variability in ASIJ number and incomplete bilateral occurrence.

Symmetry was evaluated using paired comparisons, and correlations were performed between iliac and sacral portions within the same joint (LI–LS, *n* = 21; RI–RS, *n* = 22). In specimens exhibiting bilateral ASIJ, additional comparisons were performed between left and right sides (LI–RI; LS–RS; *n* = 18 each).

To assess the relative contribution of ASIJ to CSIJ, summed AASA were expressed as proportions of the total surface area (TSA).

Associations between summed AASA and SSA, extrasynovial surface area (ESA), TSA, and body size were assessed. For body size normalization, total AASA per dog was calculated and correlated with crown–rump length (CRL).

This approach was chosen to account for variability in ASIJ presence and incomplete bilateral occurrence.

Sex‐related differences (*n* = 13 males, *n* = 12 females) were evaluated using the Mann–Whitney *U* test (*p* < 0.05). Both absolute and proportional AASA values were compared (*n* = 24), and CRL‐normalized total AASA were additionally analysed.

## 3 Results

### 3.1 SSA Shape Types

SSA shape types were categorized based on the frequency of their occurrence and the morphologies previously described in other species (Dalin and Jeffcott 1986; Jesse et al. 2017; Pallandre et al. 2020).

**FIGURE 1:**
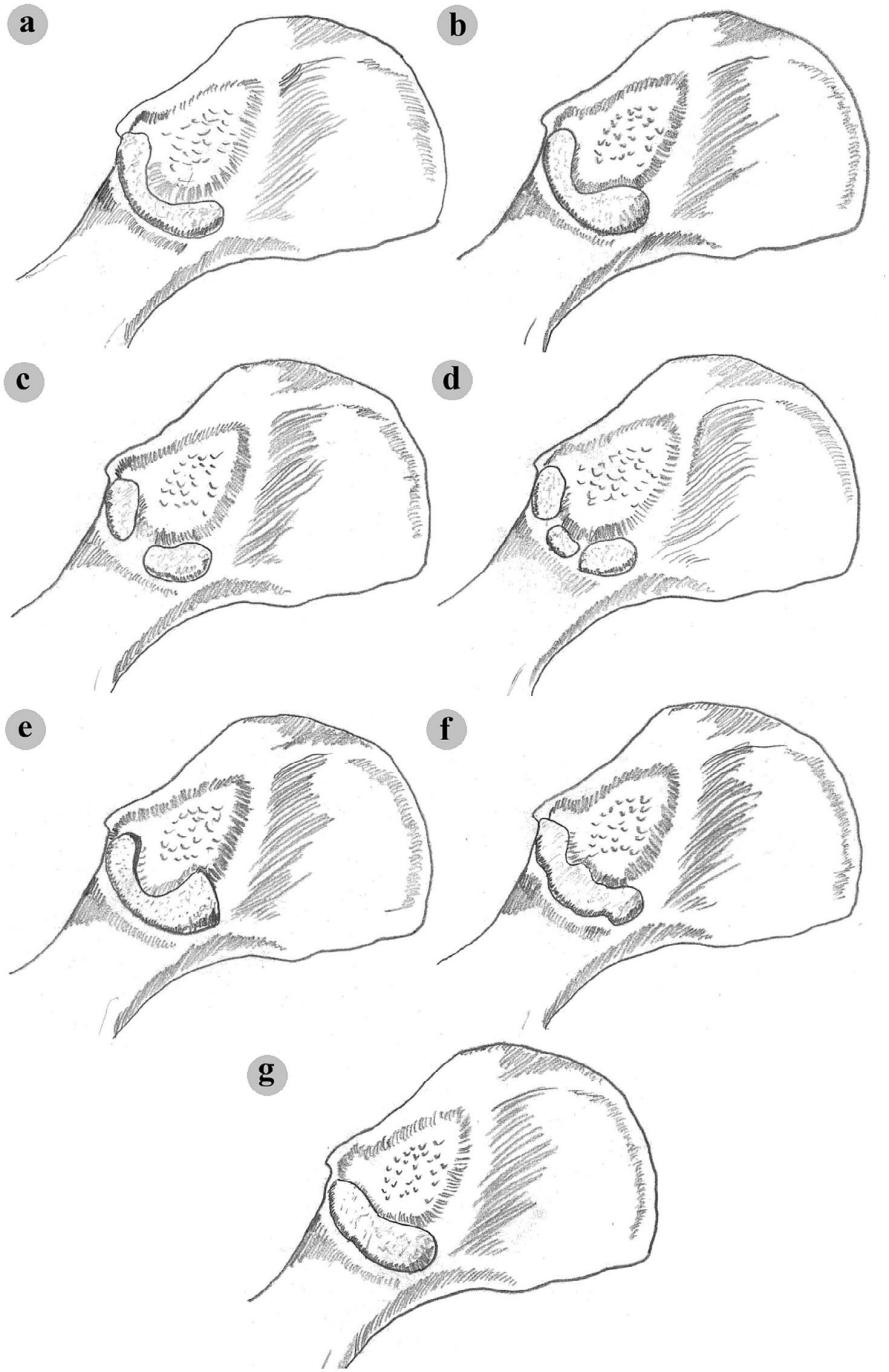
Schematic medial view of the sacropelvic surface of a left canine ilium, modified from Schaller (2018). Different shape types of the synovial surface area (SSA) of the canine sacroiliac joint (CSIJ). (a) Crescent. The synovial portion is crescent‐shaped. (b) Auricular. Similar to the crescent type, but characterized by a broader ventral than dorsal limb. (c) Bifoliate. The synovial portion is divided into two distinct lobes. (d) Trifoliate. The synovial portion is divided into three distinct lobes. (e) Phrygian cap. A broad ventral base is accompanied by a dorsally projecting limb, giving the outline the appearance of a Phrygian cap. (f) W. The outline resembles the letter *W*. (g) Spatula. The synovial portion exhibits a spatulate outline.

Table 1 and Figure 1 summarize the morphological types identified across the 116 joint portions.

**TABLE 1:** Synovial surface area shape types (*n* = 116 portions).

	Shape type						
	Auricular	Bifoliate	Phrygian Cap	W	Crescent	Spatula	Trifoliate
Left Iliac	5	7	4	5	4	4	0
Left Sacral	8	6	10	1	2	1	1
Right Iliac	7	7	4	7	2	1	1
Right Sacral	8	7	8	3	2	0	1
Total	28	27	26	16	10	6	3

Auricular (24.14%), bifoliate (23.28%), and phrygian cap–like shapes (22.41%) were the most prevalent, whereas spatula‐shaped (5.17%) and trifoliate configurations (2.59%) were rare.

Opposing SSA shapes were symmetrical in 17 of 29 CSIJ, most frequently in bifoliate and trifoliate forms. In contrast, crescent and spatula‐shaped configurations showed the lowest symmetry rates, while auricular, phrygian cap–like, and W‐shaped forms displayed a balanced distribution.

Six dogs displayed identical shapes across all four joint portions. Bilateral symmetry with differing shapes between the left and right CSIJ was observed in four cases, whereas unilateral symmetry was present in 14 dogs and complete asymmetry in five dogs.

Regional analysis revealed only minor compartment‐specific tendencies, without consistent patterns. Across all comparisons, SSA size followed a consistent hierarchy: W‐shaped forms exhibited the largest mean surface area (313.12 ± 96.19 mm^2^), followed by bifoliate (272.52 ± 221.45 mm^2^) and auricular shapes (248.50 ± 89.02 mm^2^). Phrygian cap‐like (198.36 ± 72.28 mm^2^) and crescent configurations (195.53 ± 91.32 mm^2^) showed intermediate values, whereas spatula‐shaped forms consistently displayed the smallest SSA (105.25 ± 41.62 mm^2^). These patterns were consistent across sides, joint portions, and compartments, with no evidence of clustering related to breed, body size, or sex.

### 3.2 NFOC of the Sacral SSA

**FIGURE 2:**
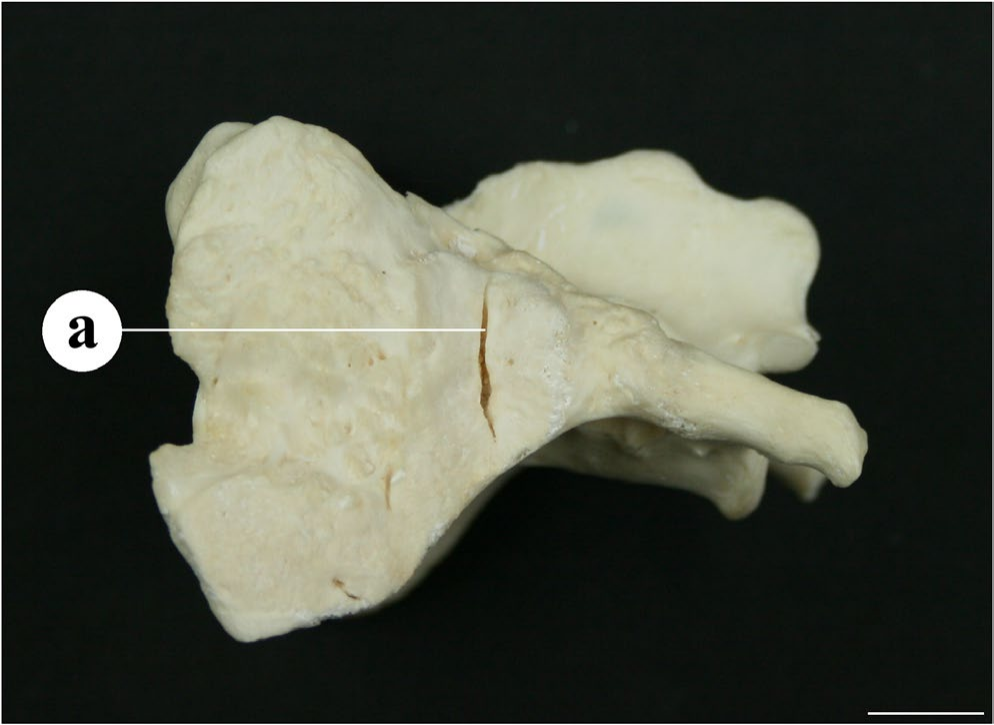
Lateral view of a left canine sacrum. (a) Cleft between non‐fused ossification centers (NFOC) at the synovial surface area (SSA). Scale bar: 1 cm.

The criteria for identifying NFOC included a partial or complete interruption or folding of the sacral SSA within its caudal third at the transition between the sacral wing and the lateral part of the sacrum. This was accompanied by a distinct change in surface inclination between the cranial and caudal portions and the formation of a concave depression (Figure 2).

NFOC were identified in 16 out of 58 CSIJ (27.59%) or in 10 out of 29 specimens (34.48%), with an equal distribution on the left and right sides (eight CSIJ on each side). In all affected joints, the typical craniolateral‐to‐caudomedial orientation of the SSA was disrupted. In the caudal third, the orientation consistently shifted to a craniomedial‐to‐caudolateral alignment. In each case, the iliac surface exhibited a corresponding adaptive change in inclination towards a craniomedial‐to‐caudolateral orientation.

On the left sacral wing, this configuration was observed in four out of eight joints with joint space formation, whereas on the right sacral wing it occurred in five out of eight joints. In the remaining joints, an almost vertical demarcation between the cranial and caudal regions was consistently present.

The caudal SSA portions measured between 46.44 ± 18.12 mm^2^ and 68.89 ± 49.73 mm^2^ on average. When normalized to TSA, these areas were relatively larger on the sacral wing compared with the iliac surface (19.99% vs. 16.70%).

No correlations with breed or sex were detected. However, NFOC were detected exclusively in dogs with a crown–rump length (CRL) exceeding 740 mm, with the exception of a single French Bulldog (CRL: 530 mm).

### 3.3 Accessory Sacroiliac Joint

#### 3.3.1 Morphological Features

In 43 out of 58 examined CSIJ, a total of 62 ASIJ were identified adjacent to the SSA. ASIJ were present in 21 out of 29 left and 22 out of 29 right CSIJ, indicating an overall symmetrical distribution (30 ASIJ in the left CSIJ and 32 in the right).

Among the examined joints, 29 out of 58 CSIJ exhibited a single ASIJ, nine joints had two ASIJ, and five joints had three ASIJ. The distribution of single, double, and triple ASIJ did not differ between iliac and sacral sides.

Bilateral occurrence was observed in 18 out of 29 dogs, whereas only four CSIJ lacked ASIJ entirely on both sides.

A total of six anatomical localizations were identified, all situated dorsal to the SSA within the region of the iliac and sacral tuberosities. The most common location was at the caudodorsal margin of the CSIJ (*n* = 32), where the sacropelvic surface of the iliac wing articulates caudomedial to the cranial dorsal iliac spine with the craniodorsal aspect of the sacral tuberosity. Immediately cranial to this region, craniodorsal articulations were observed (*n* = 8).

**FIGURE 3:**
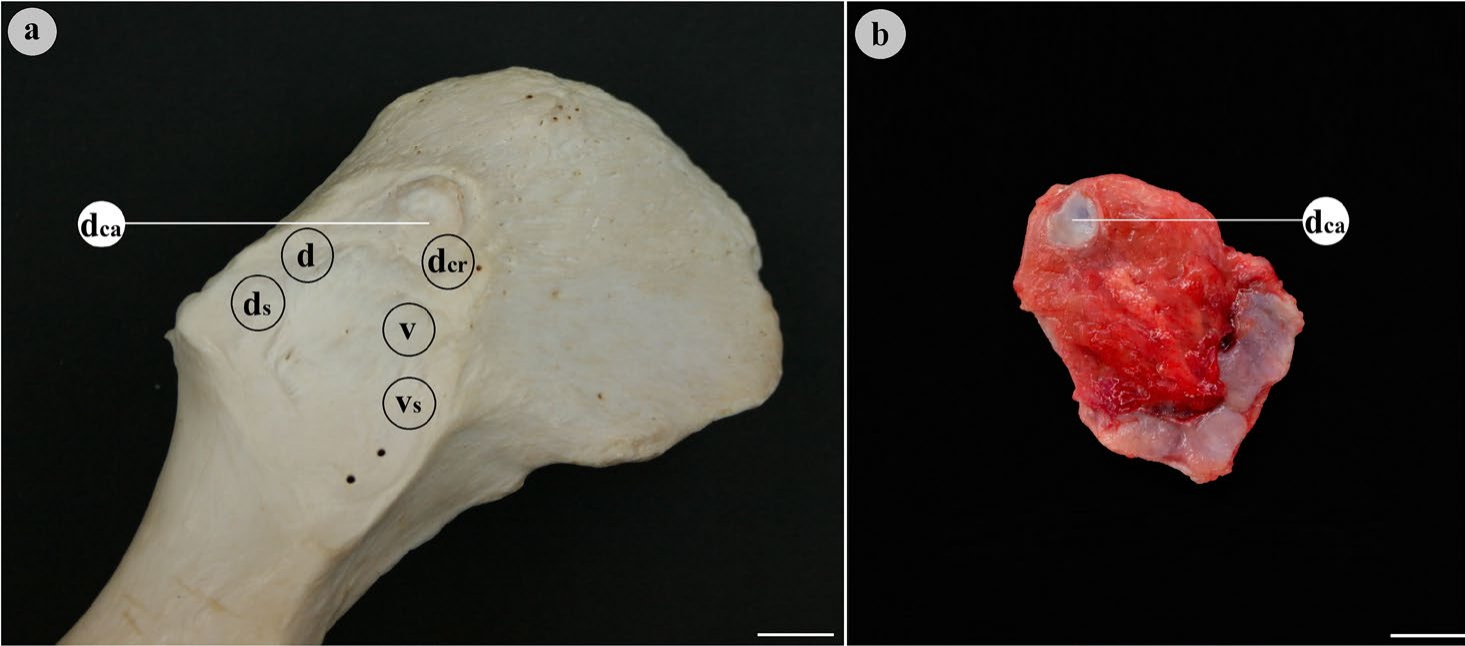
(a) Medial view of the sacropelvic surface of a left canine ilium illustrating different localizations of accessory sacroiliac joints (ASIJ). d, Dorsal; dca, Caudodorsal; dcr, Craniodorsal; ds, Dorsal (synovial extension); v, Ventral; vs, Ventral (synovial extension). Scale bar: 1 cm. (b) Lateral view of a left sacral wing following dissection. dca, Caudodorsal accessory sacroiliac joint (ASIJ). Scale bar: 1 cm.

Less frequently, ASIJ were located ventrally at the margin of the ESA, near the first dorsal sacral foramen (ventral *n* = 3; dorsal *n* = 5). In addition, ASIJ were observed as extensions of the SSA at both its cranioventral (*n* = 4) and caudodorsal limbs (*n* = 10) (Figure 3).

The iliac and sacral components of the ASIJ were congruent, forming either flat or concave–convex articulations. Focal osseous formations were present in 27.42% of all ASIJ. The AASA were predominantly round to oval in shape, occasionally tapering, and nearly half (48.39%) showed an orientation deviating from that of the primary joint.

No apparent associations were observed between the presence of ASIJ and sex, body size, or breed.

#### 3.3.2 Histology of ASIJ Cartilage

**FIGURE 4:**
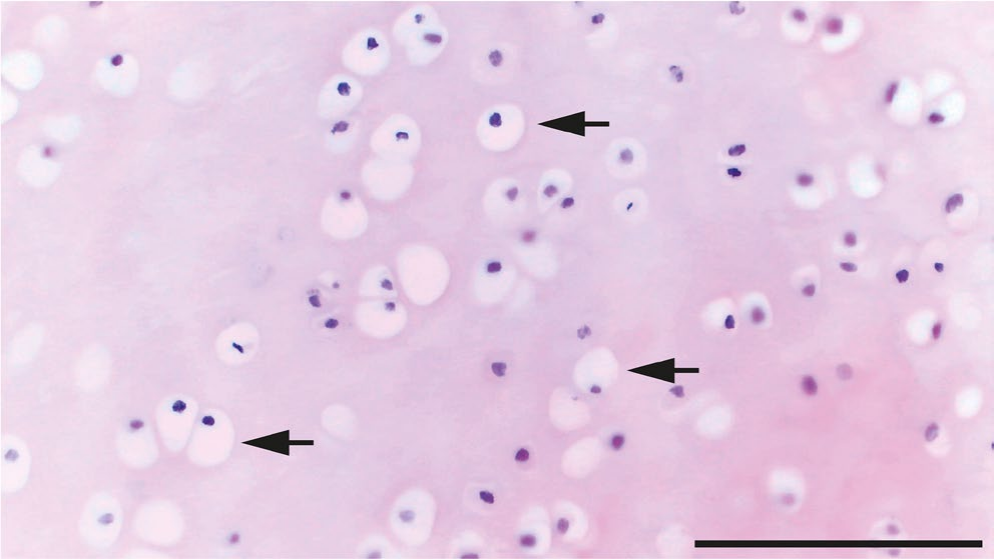
Histological appearance of the hyaline cartilage lining a left iliac caudodorsal canine accessory sacroiliac joint (ASIJ), stained with haematoxylin & eosin (H&E). Chondrocytes are arranged in chondrons. Scale bar: 100 μm (40×).

The superficial layer of all four caudodorsal ASIJ of dog no. 25 consisted of hyaline cartilage (Figure 4). Histologically, the cartilage exhibited a homogeneous extracellular matrix with mild eosinophilic staining. Chondrocytes were located within lacunae and displayed basophilic nuclei, staining dark blue to purple, while the cytoplasm was faintly eosinophilic. The chondrocytes were observed either individually or arranged in small clusters, referred to as isogenous groups. No histological features indicative of fibrocartilage or elastic cartilage were detected in any sample.

#### 3.3.3 Morphometric Features of ASIJ

The mean surface area of all AASA was 42.83 mm^2^. The smallest AASA (4.20 mm^2^) was observed at the craniodorsal aspect of LI in a male German Shepherd Dog crossbreed (No. 9), whereas the largest (260.80 mm^2^) occurred caudodorsally in RI of a female Bernese Mountain Dog (No. 30).

In dogs exhibiting one or more ASIJ, the summarized mean value of AASA per individual was 61.81 mm^2^.

With respect to anatomical localization, the largest AASA were found caudodorsally (52.48 mm^2^), followed by dorsal (47.22 mm^2^), dorsal to the SSA (39.89 mm^2^), and ventral to the SSA (33.10 mm^2^). The smallest AASA were found in ventral (17.48 mm^2^) and craniodorsal locations (14.46 mm^2^; Figure 3).

Strong positive correlations were observed between iliac and sacral AASA within both left and right CSIJ (*ρ* = 0.9, *p* < 0.001). In specimens with bilateral AASA, moderate correlations were identified between LI and RI as well as between LS and RS (*ρ* ≤ 0.577, *p* ≤ 0.004).

No significant difference was detected between LI and LS (*W* = 171, *p* = 0.055), whereas RI was significantly larger than RS (*W* = 210, *p* = 0.007). In bilateral cases, iliac AASA were significantly larger on the right side (*W* = 31, *p* = 0.016), while sacral portions did not differ (*W* = 55, *p* = 0.196).

Overall, AASA contributed a mean value of 7.25% to TSA, with proportions of 5.78% (LI), 6.37% (LS), 8.36% (RI), and 8.48% (RS). Pairwise comparisons of proportional contributions yielded correlation patterns consistent with those observed in symmetry analysis.

**FIGURE 5:**
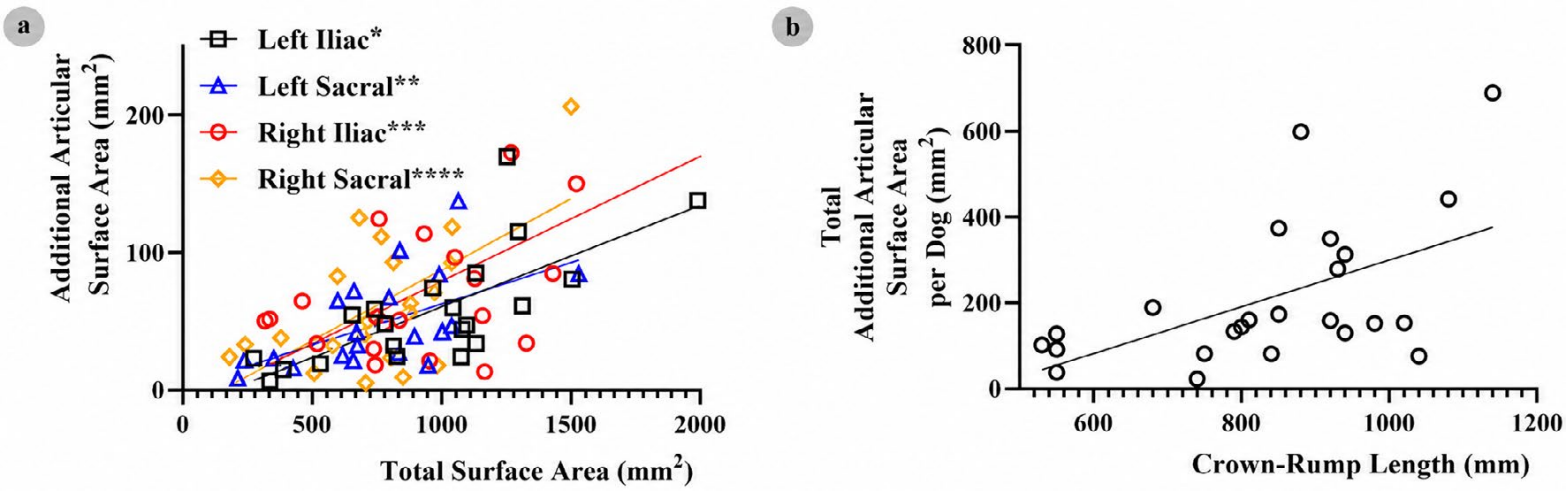
(a) Additional articular surface area (AASA) to total surface area (TSA)‐relation (*ρ = 0.755, *p* < 0.001; **ρ = 0.64, *p* = 0.002; ***ρ = 0.436, *p* = 0.044; ****ρ = 0.614, *p* = 0.002). (b) Total AASA per dog to crown‐rump length (CRL)‐relation (*ρ* = 0.523, *p* = 0.009). Solid lines represent linear regression, respectively.

AASA showed significant positive correlations with TSA across all joint portions (*ρ* = 0.436–0.755, *p* = < 0.001–0.044; Figure 5a) and even stronger correlations with ESA (*ρ* = 0.470–0.831, *p* = < 0.001–0.028). No significant correlations were observed with SSA, although a weak trend was noted for LI (*ρ* = 0.403, *p* = 0.071). The total AASA per dog correlated moderately with CRL (*ρ* = 0.523, *p* = 0.009; Figure 5b).

No sex‐specific differences in AASA morphometry were detected.

## 4 Discussion

### 4.1 SSA Shape Types

This study demonstrates that SSA morphology in dogs exhibits considerable variability beyond the commonly assumed auricular or crescent‐shaped configurations; only 38 of the 116 examined surfaces conformed to the classical patterns. The shape classifications applied were based on variants previously described in other species (Dalin and Jeffcott 1986; Jesse et al. 2017; Pallandre et al. 2020).

The greatest morphological similarity was observed between dogs and felids, as both species display bifoliate, spatula‐shaped, and phrygian cap–like configurations. In contrast, W‐shaped and trifoliate configurations were identified exclusively in dogs and, to the best of our knowledge, have not been reported in other species to date.

In humans, auricular SSA are more frequently observed in individuals without sacroiliac (SI) pain, whereas crescent‐shaped surfaces are more commonly associated with SI pain. This observation suggests a potential relation between SSA morphology and the development of SI pain. Different joint morphologies are thought to lead to distinct patterns of load distribution and force transmission. Compared with more oval configurations, crescent‐shaped surfaces provide a smaller area for load transfer. Consequently, a reduced SSA may shift a greater proportion of mechanical forces to adjacent stabilizing structures, such as the ligamentous apparatus. This increases mechanical stress and promotes degenerative changes (Jesse et al. 2017).

Accordingly, dogs exhibiting smaller SSA—particularly those associated with phrygian cap, crescent, or spatula shapes—may be more susceptible to degenerative alterations and, consequently, to the development of SI pain. Moreover, SSA lacking congruent or opposing surface symmetry, as frequently observed in crescent and spatula configurations, may experience a further reduction in effective contact area. This reduction could lead to an increased relative load per unit surface area and thus elevated mechanical stress on the CSIJ, potentially resulting in altered load distribution and subsequent biomechanical imbalance, concepts that have similarly been discussed in the context of the human knee (Gannon et al. 2021; Imhauser et al. 2013). Taken together, dogs, similar to cats, horses, and humans, exhibit substantial variability in SSA morphology (Dalin and Jeffcott 1986; Jesse et al. 2017; Pallandre et al. 2020). If concepts derived from human medicine are cautiously applied, joint configurations characterized by smaller surface areas and reduced bilateral or opposing surface symmetry may be particularly prone to joint alterations.

### 4.2 NFOC of the Sacral SSA

Concave depressions characterized by clefting or folding, accompanied by a change in inclination of the caudal third of the SSA, correspond to four OC associated with the transverse processes of S1 and S2, as well as two additional isolated OC located at their ventral aspects. Under physiological conditions, these OC typically fuse completely by approximately 25 weeks of age, with earlier fusion reported in smaller breeds. Interruptions of this fusion process represent NFOC, which have been described as a reactive response to mechanical overload of the immature SSA. By increasing local interlocking between opposing joint surfaces, NFOC have been suggested to contribute to joint stabilization. As isolated OC may be absent in small breeds, this phenomenon appears to occur predominantly in large dogs. Canine NFOC can be detected as early as 24 weeks of age and may occasionally persist into adulthood (Breit and Künzel 2001b).

Similar findings in humans have primarily been reported in adolescents and young adults, albeit with a markedly lower prevalence (Götz et al. 1993; Green et al. 2008; Prassopoulos et al. 1999). In this context, NFOC are generally considered to be non‐pathological (Götz et al. 1993). This interpretation is supported by a case report that excluded them as a cause of SI pain, as their presumed lifelong presence makes an onset of pain later in life unlikely (Green et al. 2008).

Nevertheless, complete fusion may result in osseous prominences of the SSA, which could contribute to degenerative changes. In imaging studies, NFOC may initially mimic fractures (Prassopoulos et al. 1999), highlighting the importance of reliable differentiation from fractures and other degenerative joint alterations. Axial CT appears to be the most suitable modality for this purpose (Götz et al. 1993), where NFOC typically present as triangular osseous bodies within the anterosuperior joint space (Prassopoulos et al. 1999).

In summary, NFOC of the canine sacral SSA appear to be more prevalent than in humans. Although they are not considered a direct cause of SI pain, their accurate identification is crucial to avoid diagnostic misinterpretation. Moreover, they may even contribute functionally to the stabilization of the CSIJ.

### 4.3 Accessory Sacroiliac Joint

The iliac and sacral tuberosities, which together form the osseous basis of the ESA, are incongruent and covered by fibrocartilage (Evans and Lahunta 2013). Consequently, the CSIJ was classified as a composite joint, comprising a diarthrodial component in the caudoventral region and a symphyseal component in the craniodorsal region (Gensicke et al. 2026).

#### 4.3.1 Morphological Features of ASIJ

Congruent AASA within the ESA were identified more frequently than expected. These surfaces were either flat or concave–convex in configuration and were covered by hyaline cartilage.

Comparable joint variants are less frequent in humans but have been reported in anatomical dissection and imaging studies, with prevalences ranging from 6.8% to 50% (CT 1.7%–25.8%, MRI 5%–11%). These structures are referred to as ASIJ and are defined as AASA located dorsal to the primary diarthrodial joint (Cihan et al. 2023; Tok Umay and Korkmaz 2020; Trentadue et al. 2023; Wittram and Whitehouse 1995).

ASIJ may occur bilaterally (4.4%–53%) in humans (Ehara et al. 1988; El Rafei et al. 2018; Prassopoulos et al. 1999; Valojerdy and Hogg 1990), a pattern likewise observed in the present canine study, in which 62% of cases showed bilateral involvement, predominantly with symmetrical distribution. Notably, approximately one quarter of dogs had multiple ASIJ within a single CSIJ, a feature that has not been reported in humans (Valojerdy and Hogg 1990).

The most commonly reported location of ASIJ in humans corresponds largely to the rare canine ventral ASIJ (4.76%). More ventrally positioned human ASIJ resemble the dorsoventral variants identified in dogs (11.29%) (Demir et al. 2007; Ehara et al. 1988; Prassopoulos et al. 1999; Trentadue et al. 2023).

In contrast, four additional ASIJ localizations were identified in dogs, most frequently at the caudodorsal joint margin (51.61%). Further variants included extremely dorsal ASIJ (9.68%) as well as extensions exceeding the regular SSA ventrally (6.45%) and dorsally (16.13%).

In dogs, force transmission across the CSIJ is influenced by the horizontally oriented trunk, propulsion from the hindlimbs via the pelvis to the spine (Prieur 1980), and additional rotational forces during locomotion (Breit and Künzel 2001b). In contrast, axial compression predominates in the upright posture of humans. This more complex and dynamic loading environment in quadrupeds may promote a more frequent and more distributed adaptive formation of ASIJ compared to the predominantly vertically loaded human SIJ.

#### 4.3.2 Morphometric Features of ASIJ

Canine ASIJ dimensions showed considerable variability, ranging from 14.46 to 52.48 mm^2^, corresponding to 5.78 to 8.48% of the TSA. In humans, reported AASA fall within a comparable range, with mean values of approximately 47 ± 36 mm^2^ (Valojerdy and Hogg 1990), although considerably larger surface areas, ranging from 78.00 to 314.00 mm^2^, have also been described (Hadley 1950).

The observed variability in ASIJ sizes appears to be primarily attributable to size‐dependent allometric scaling, as indicated by correlations with body size (CRL) and joint dimensions (ESA and TSA). In addition, load‐dependent biomechanical adaptation in response to individual mechanical demand may contribute further to the broad size spectrum.

In contrast to findings in humans (Valojerdy and Hogg 1990), no correlation could be demonstrated between AASA and SSA in dogs. This discrepancy may reflect the same species‐specific biomechanical conditions as previously discussed in the context of gait variation.

Iliac and sacral AASA were significantly correlated, as were right and left AASA in dogs exhibiting bilateral ASIJ. LI AASA tended to be larger than the LS AASA. On the right side, RI were significantly larger than the RS AASA. This asymmetry is consistent with previously reported morphometric patterns of the CSIJ (Gensicke et al. 2026).

Sacral ASIJ dimensions did not differ significantly between sides. The reason for the larger RI AASA compared with LI cannot be conclusively determined based on the present data and may reflect individual loading patterns or anatomical variability.

ASIJ morphology appeared to be independent of the morphology of the main joint. This observation supports the hypothesis that the ASIJ develops independently of the CSIJ and may fulfil a distinct biomechanical role within the SI complex.

#### 4.3.3 Classification and Aetiology of the ASIJ

The identification of hyaline cartilage and a distinct joint space in the present study supports the classification of the ASIJ as a diarthrosis, specifying previous human findings (Ehara et al. 1988). Accordingly, the ASIJ represents histologically distinct structures within the otherwise predominantly fibrocartilaginous ESA (Gensicke et al. 2026). Whereas the surrounding ESA is characterized by dense fibrous connective tissue with variable amounts of fibrocartilage, the ASIJ exhibits the histological features of a synovial articulation, including a joint cavity lined by opposing hyaline cartilaginous surfaces. These findings indicate that the ASIJ should not be interpreted merely as a focal fibrocartilaginous specialization of the ESA, but rather as a separate diarthrodial articulation.

Although our study population consisted predominantly of adult individuals, diarthrodial ASIJ were also identified in one adolescent specimen (11‐month‐old female Bernese Mountain Dog), suggesting that canine ASIJ can be congenital.

A congenital aetiology has also been suggested for humans (Ehara et al. 1988; Trotter 1937).

However, most human studies assume an acquired origin of ASIJ. Their prevalence and number increase with age (Prassopoulos et al. 1999; Trentadue et al. 2023), body weight, and parity (Prassopoulos et al. 1999), suggesting that mechanical stress may promote their development. This hypothesis is further supported by studies reporting the absence of ASIJ in individuals younger than 15 years (Rixey et al. 2021).

The osseous formations observed in the present study may correspond to the projections described by Demir et al. (2007), Ehara et al. (1988), and Prassopoulos et al. (1999) (reported a prevalence of 14.71%) and could represent degenerative remodelling patterns, thereby supporting an acquired origin in dogs.

#### 4.3.4 Clinical Implications of the ASIJ

In humans, ASIJ are the most frequent SIJ variant and have been consistently linked to degenerative change (Demir et al. 2007; El Rafei et al. 2018) as well as low back pain (Hadley 1950; Kang et al. 2017; Prassopoulos et al. 1999; Rosa Neto et al. 2009; Slobodin et al. 2017; Toussirot et al. 2018). Sclerosis, osteophyte formation, and ankylosis occur more frequently in ASIJ than in the main joint and are reported to be symptomatic in most affected individuals. These alterations have been attributed, at least in part, to the discordant orientation of the ASIJ relative to the SIJ (Hadley 1950; Prassopoulos et al. 1999), resulting in altered load transmission and increased mechanical stress (Hadley 1950).

The present study demonstrates that ASIJ are considerably more prevalent in dogs than reported in humans and may occur as multiple structures within a single CSIJ. Because the ASIJ possess the histological characteristics of a diarthrosis, they are expected to permit controlled motion while simultaneously transmitting forces. Their presence may therefore modify the distribution of mechanical loads across the SI region by providing additional focal contact areas, particularly in dogs exhibiting large or multiple ASIJ or additional morphological risk factors such as reduced SSA or altered joint congruency.

These findings highlight the potential clinical relevance of canine ASIJ and emphasize the importance of recognizing such variants in anatomical and radiological evaluations of the canine SI region. Further studies integrating imaging, biomechanical modelling, and clinical correlation are required to clarify their role in pain, degenerative processes, and functional impairment in dogs.

## 5 Conclusion

This study demonstrates that the CSIJ exhibits substantial morphological variability that extends beyond the primary articulation. Seven SSA shape types, NFOC, and ASIJ were identified as common variants rather than rare anomalies. The ASIJ were markedly more prevalent in dogs than has been reported in humans and were often observed bilaterally or as multiple structures within a single joint. The presence of hyaline cartilage lining the ASIJ supports their classification as true diarthrodial articulations. While clinical correlation was not available, these findings suggest that these variants may function as independent joint structures and therefore influence joint biomechanics and load distribution. Therefore, recognition of this anatomical diversity is therefore essential for the accurate interpretation of imaging findings and provides a morphological foundation for future biomechanical and clinical investigations of the CSIJ.

## Author Contributions

**Janek Gensicke:** writing – original draft, visualization, validation, project administration, methodology, investigation, formal analysis, statistical analysis, data curation, conceptualization. **Mirjam Kalusa:** writing – review and editing, validation, supervision. **Thomas Grochow:** writing – review and editing, validation, supervision, visualization. **Rebecca L. Tartsch:** writing – review and editing, validation. **Alexandra S. Klick:** writing – review and editing, validation. **Christoph K. W. Mülling:** writing – review and editing, validation, resources. **Simone A. Fietz:** writing – review and editing, validation, resources. **Nicole Röhrmann:** writing – review and editing, validation, supervision, resources, project administration, conceptualization.

## Funding

This work was supported by Dr. Heidi und Karl‐Heinz Kübler Stiftung.

## Conflicts of Interest

The authors declare no conflicts of interest.

## Supporting information


**Table S1:** Overview of dissected specimens.

## Data Availability

The data that support the findings of this study are available from the corresponding author upon reasonable request.
