# Supplementary material for: The Canine Sacroiliac Joint: 2. Common Surface Variants
Source: Anat Histol Embryol. 2026 Jul 27;55(5):e70159. doi: 10.1111/ahe.70159 (PMC13408332; doi:10.1111/ahe.70159)
Supplement: Supplementary file 1 — Table S1: Overview of dissected specimens. [file AHE-55-e70159-s001.docx]

**Supplementary Table 1 | Overview of dissected specimens**

| No. | Sex | Breed | Body Weight (kg) | BCS | CRL (mm) | Age |
| --- | --- | --- | --- | --- | --- | --- |
| 7 | male | Irish Setter | 27.20 | 3.5 | 940 | adult |
| 8 | female | Boxer | 21.30 | 4 | 940 | adult |
| 9 | male | German Shepherd-Mix | 23.30 | 4 | 880 | adult |
| 10 | male | German Shepherd-Husky-Mix | 17.10 | 5 | 880 | adult |
| 11 | male, neutered | Shiba Inu-Mix | 17.35 | 5.5 | 680 | adult |
| 12 | male | Beagle-Mix | 12.75 | 5.5 | 660 | adult |
| 13 | female, spayed | German Shepherd-Mix | 11.65 | 4 | 750 | adult |
| 14 | female | German Shepherd-Mix | 25.10 | 4 | 920 | adult |
| 15 | male | Cane Corso | 45.60 | 5 | 930 | six y |
| 16 | female | Labrador Retriever | 38.90 | 7.5 | 740 | adult |
| 17 | male, neutered | Poodle-Maltese-Mix | 7.70 | 5 | 550 | adult |
| 18 | male | Greyhound-Mix | 28.10 | 4 | 1,040 | adult |
| 19 | female | Labrador Retriever | 33.30 | 6 | 1,020 | 13 y |
| 20 | female | Mix | 14.90 | 5 | 790 | 16 y |
| 21 | female | Rehpinscher | 7.70 | 1 | 550 | adult |
| 22 | female, spayed | Mix | 7.70 | 5 | 550 | 12 y |
| 23 | male, neutered | Mix | 23.10 | 6.5 | 800 | adult |
| 24 | female, spayed | French Bulldog | 12.65 | 5 | 530 | adult |
| 25 | male | Bernese Mountain Dog | 38.00 | 5 | 810 | 11 m |
| 27 | male | Golden Retriever-Mix | 27.20 | 4 | 850 | adult |
| 28 | female | unknown | 21.30 | 4 | 850 | adult |
| 29 | female | Husky | 34.60 | 5 | 980 | adult |
| 30 | female | Bernese Mountain Dog | 44.00 | 5 | 1,140 | adult |
| 31 | male, neutered | Afghan Hound | 24.30 | 3 | 1,080 | adult |
| 32 | male, neutered | Bernese Mountain Dog-Mix | 28.80 | 5 | 920 | adult |
| 33 | male, neutered | Labrador Retriever | 29.20 | 5 | 940 | 13 y |
| 34 | male | unknown | 14.95 | 4 | 790 | adult |
| 35 | male | unknown | 22.20 | 5 | 850 | adult |
| 36 | male, neutered | German Shepherd-Husky-Mix | 27.00 | 6 | unknown | adult |
